# Immunocompromised Travelers: Demographic Characteristics, Travel Destinations, and Pretravel Health Care from the U.S. Global TravEpiNet Consortium

**DOI:** 10.4269/ajtmh.15-0185

**Published:** 2015-11-04

**Authors:** Brian S. Schwartz, Jessica Rosen, Pauline V. Han, Noreen A. Hynes, Stefan H. Hagmann, Sowmya R. Rao, Emily S. Jentes, Edward T. Ryan, Regina C. LaRocque, the Global TravEpiNet Consortium

**Affiliations:** Division of Infectious Diseases, University of California, San Francisco, California; Division of Infectious Diseases and Travel Medicine, Georgetown University, Washington, District of Columbia; Division of Global Migration and Quarantine, Centers for Disease Control and Prevention, Atlanta, Georgia; Johns Hopkins Travel and Tropical Medicine, Division of Infectious Diseases, Johns Hopkins School of Medicine, Baltimore, Maryland; Division of Pediatric Infectious Diseases, Bronx Lebanon Hospital Center, Bronx, New York; Department of Pediatrics, Albert Einstein College of Medicine, Bronx, New York; Department of Quantitative Health Sciences, University of Massachusetts Medical School, Worcester, Massachusetts; Center for Health Quality, Outcomes and Economics Research, Bedford Veterans Affairs Medical Center, Bedford, Massachusetts; Travelers' Advice and Immunization Center, Massachusetts General Hospital and Harvard Medical School, Boston, Massachusetts

## Abstract

An increasing number of immunocompromised individuals are pursuing international travel, and a better understanding of their international travel patterns and pretravel health care is needed. We evaluated the clinical features, itineraries, and pretravel health care of 486 immunocompromised international travelers seen at Global TravEpiNet sites from January 2009 to June 2012. We used bivariate analyses and logistic regressions using random intercept models to compare demographic and travel characteristics, vaccines administered, and medications prescribed for immunocompromised travelers versus 30,702 immunocompetent travelers. Immunocompromised travelers pursued itineraries that were largely similar to those of immunocompetent travelers, with nearly one-third of such travelers visiting countries with low human development indices. Biological agents, including tumor necrosis factor blockers, were commonly used immunosuppressive medications among immunocompromised travelers. A strong collaboration between travel-medicine specialists, primary care doctors, and specialist physicians is needed to prepare immunocompromised people for international travel. Incorporating routine questioning and planning regarding travel into the primary care visits of immunocompromised people may be useful.

## Introduction

There were more than 60 million trips to foreign countries undertaken by U.S. citizens in 2013.^1^ Travel to countries with developing economies, such as those in Africa and Asia, has increased over the past 20 years.^2^ Concurrently, there has been an increase in the number of people living with immune-compromising conditions, such as those receiving disease-modifying medications that suppress one or more immune pathways, recipients of transplanted organs or stem cells,^3^ and persons with late-stage human immunodeficiency virus (HIV) infection.^4^ In a recent survey of U.S. solid-organ transplant recipients, 27% reported travel outside the United States or Canada.^5^ Similarly, a reported 20–46% of HIV-infected patients travel internationally.^6^–^8^

A pretravel health consultation is particularly important for the immunocompromised traveler, but may be challenging for practitioners without extensive experience with immunocompromised patients. For instance, immunosuppression influences clinical decision making about whether a traveler should receive selected vaccines and also may affect the level of immune protection achieved after immunization with both routine and travel-related vaccines.^9^ Immunocompromised people are at higher risk for travel-related complications and have higher rates of hospitalization when travel-related illness occurs.^10^,^11^ Pretravel planning, including ensuring an adequate supply of routine medications and consideration of medical evacuation insurance, is therefore particularly important for these travelers.

A better understanding of the travel patterns and pretravel health care of immunocompromised people is needed to inform specific guidance for such travelers and medical providers who care for them. We evaluated a large cohort of immunocompromised travelers who obtained pretravel health advice in Global TravEpiNet (GTEN), a consortium of U.S. practices that provide pretravel care to international travelers. Our goals were to describe the demographics, itineraries, and pretravel care of immunocompromised travelers who sought pretravel health advice, to compare these characteristics with immunocompetent travelers at these sites, and to identify areas in which the pretravel preparation of this population could be improved.

## Methods

### Consortium description.

GTEN is a U.S. Centers for Disease Control and Prevention (CDC)-supported consortium of clinical practices that provide pretravel health care, as previously described.^12^ In brief, GTEN sites are distributed across the United States and include academic practices, health-care consortia, health-maintenance organizations, pharmacy-based clinics, private practices, and public health clinics. Human subject advisors at each participating site reviewed and approved or exempted the collection and subsequent analyses of the de-identified data.

### Data collection and description.

Clinicians collected de-identified data on all people seen for pretravel consultation at 21 participating sites from January 2009 through June 2012 by using a secure internet tool. For each unique clinic visit, travelers provided details about their reasons for seeking consultation, medical history, number of itineraries, countries of planned travel, dates of travel, planned accommodations, purpose(s) of travel, setting(s) of travel, and planned activities. Travelers selected one or more of the following purposes for their trips: leisure, business, returning to region of origin of self or family to visit friends and relatives, adoption, providing medical care, receiving medical care, research/education, nonmedical service work, missionary work, military service, adventuring, attending large gatherings or events, or other activities. Clinicians verified and further clarified, as needed, the information provided by travelers and entered additional data on immunization history, health advice provided, vaccines administered, and medications prescribed during the pretravel encounter. If a traveler had an indication for a vaccine according to the Advisory Committee on Immunization guidelines that were current at the time of the clinic visit, but the vaccine was not administered, the clinician was required to provide a reason for not administering the vaccine; available options included preexisting immunity, vaccine not indicated, referred to primary care provider for vaccination, patient declined, medical contraindication, insufficient time, or vaccine not available.

### Definition of immunocompromised travelers.

We defined an immunocompromised traveler as a person with one or more of the following conditions: human immunodeficiency virus/acquired immunodeficiency syndrome (HIV/AIDS), organ or stem cell transplant, hematologic malignancy, thymectomy, splenectomy, sickle cell anemia, or current receipt of an immunosuppressive medication. Immunosuppressive medications were defined as corticosteroids (equivalent to ≥ 20 mg prednisone/day), methotrexate, biological treatment agents that may suppress one or more immune pathways (including tumor necrosis factor [TNF] blockers, rituximab, and other potentially immunosuppressive biologic therapies), calcineurin inhibitors (cyclosporine, tacrolimus), mycophenolate mofetil, antimetabolites (azathioprine, 6-mercaptopurine), sirolimus, leflunomide, hydroxyurea, alkylating agents, and immunosuppressive cancer chemotherapy. We excluded patients from the analysis if we were unable to determine the level of immunosuppression based on available data (e.g., a reported non-hematological malignancy without treatment details or steroids of unknown dose). All other travelers were classified as immunocompetent.

### Classification of destination countries and visiting friends and relatives travelers.

We classified destination countries in accordance with the 2011 U.N. Human Development Index.^13^ In accordance with the CDC definition of the term,^9^ we defined a VFR (visiting friends and relatives) traveler as a person who was traveling to a country with a low or medium human development score and who selected “traveling to region of origin of self or family to visit friends or relatives” as his or her purpose of travel.

### Data analysis.

Data analyses were performed by using SAS 9.3 (SAS Institute, Cary, NC) and Excel 2010 (Microsoft Corporation, Redmond, WA). Bivariate analyses were done by using χ^2^ test for categorical variables and Wilcoxon rank-sum test for continuous variables. Logistic regressions were used to test for associations between demographics, travel characteristics, medicines, and immunosuppressed status. Because of possible clinic variation, random intercept models using clinic as the random effect with a correction for a small number of clusters were used.^14^ Statistical significance was determined at a two-sided *P* value of 0.05 for all tests.

## Results

During the study period, 32,099 travelers were seen at 21 GTEN sites. Because the immune status of 911 people could not be determined, these travelers were excluded from the analysis. For the remaining 31,188 travelers included in the analysis, we classified 486 (1.6%) as immunocompromised and 30,702 as immunocompetent (98%). Use of an immunosuppressive medication, HIV/AIDS, and receipt of a solid-organ transplant were the most common immmunocompromising conditions in our study cohort (Table 1). Among the 110 HIV-positive travelers in the study, 71 (65%) had a CD4 count above 500 cells/mm^3^. Corticosteroids and TNF inhibitors were the most commonly used immunosuppressive medications (Table 1).

Immunocompromised travelers were older than the general population of travelers (Table 2); 61 (13%) were over the age of 65 years. India was the most frequent destination country for both immunocompromised and immunocompetent travelers. Immunocompromised travelers traveled for a similar length of time as immunocompetent travelers (median, 14 days); both groups of travelers were seen a median of 25 days before departure. Thirteen percent of immunocompromised travelers were pursuing VFR travel, and 19% were staying with relatives. Immunocompromised travelers were more likely to travel on cruise ships than immunocompetent travelers (8% versus 4%, *P* < 0.001; Table 2). Immunocompromised travelers took a median of three medications, which was significantly higher than immunocompetent travelers (median of 1 medication).

We evaluated the travel characteristics of different types of immunocompromised travelers (Table 3). Few solid-organ transplant recipients traveled to countries with low U.N. human development indices. Approximately half of HIV-positive travelers (46% of those with CD4 < 500 and 51% of those with CD4 > 500) were traveling to countries with a low U.N. human development index. African countries were the most common destinations among HIV-positive travelers, whereas India was the most common travel destination among other immunocompromised people. Leisure was the most common purpose of travel for all groups, but HIV-positive travelers and recipients of solid-organ transplants were commonly identified as VFR travelers. Of people taking TNF blockers, almost one-quarter were pursuing business travel.

Overall, 93% of immunocompromised travelers were traveling to countries where malaria is wholly or partially endemic. Significantly fewer immunocompromised travelers than immunocompetent travelers who were visiting malaria-endemic countries were prescribed malaria chemoprophylaxis (281 [62%] versus 19,432 [69%], *P* = 0.001). Of the 108 immunocompromised travelers visiting countries wholly endemic for malaria, 92 (85%) received malaria chemoprophylaxis. Atovaquone/proguanil was the most commonly prescribed agent for malaria chemoprophylaxis in both immunocompromised and immunocompetent travelers. Antibiotics for the presumptive self-treatment of travelers' diarrhea were prescribed at a similar rate in the immunocompromised and immunocompetent populations (79% and 78%, respectively). Only 6% of immunocompromised travelers were prescribed prophylactic antibiotics to prevent traveler's diarrhea, which was similar to the frequency in immunocompetent travelers (7%).

**Figure 1.**
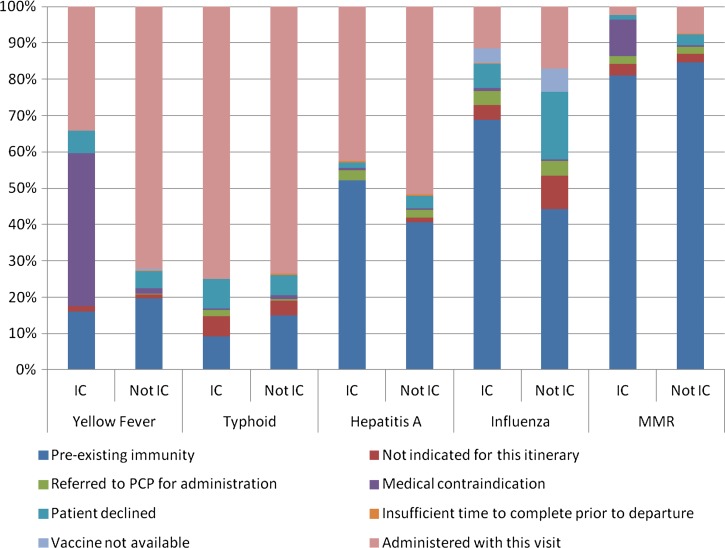
Vaccine receipt in immunocompromised travelers (IC) and immunocompetent travelers (notIC).

Among the 486 immunocompromised travelers, typhoid (75%) and hepatitis A (43%) were the most frequently administered vaccines. Yellow fever (42% of the 149 immunocompromised travelers to yellow fever-endemic countries) and measles, mumps, and rubella (10% of the 486 immunocompromised travelers) were the vaccines that clinicians most frequently considered to be contraindicated in immunocompromised travelers (Figure 1

). Fifty-one (34%) of the 149 immunocompromised travelers who were visiting yellow fever-endemic countries received the yellow fever vaccine; 29 (57%) of these travelers were HIV positive (83% of whom had a CD4 count > 500 cells/mm^3^). Four patients on TNF inhibitors received the yellow fever vaccine, and no solid-organ transplant recipients received the yellow fever vaccine. Seven percent of immunocompromised travelers, as compared with 19% of immunocompetent travelers, declined influenza vaccination that was suggested by the clinician (Figure 1).

## Discussion

Our study is the largest to date to focus on pretravel care of immunocompromised U.S. international travelers. The travelers in our study had a range of immunocompromising conditions, ranging from receipt of a solid-organ transplant to prescription of low doses of methotrexate.^9^ In general, immunocompromised travelers in our sample were pursuing similar types of travel as immunocompetent travelers, and our findings highlight that pretravel care and advice for these travelers is complex. For instance, immunocompromised people were taking multiple medications and medical contraindications to travel-related vaccination, such as yellow fever, were common.

We found that travelers receiving biological agents, such as TNF inhibitors, represented a large proportion of the immunocompromised traveling population. TNF inhibitors were among the most commonly used immunosuppressive medication in our study, likely reflecting the increasing number of indications for their use in recent years. Importantly, these medications increase the risk for severe complications of certain infections that may be related to travel, including tuberculosis, endemic mycoses, non-typhoidal salmonellosis, and leishmaniasis.^15^–^18^ Vaccine responses, particularly to influenza and pneumococcal vaccines, may be diminished in people taking TNF blockers, but vaccination is nevertheless recommended.^19^–^21^ Live-virus vaccination is contraindicated in people receiving immune-modulating agents, such as TNF blockers. Travel medicine practitioners and others caring for immunocompromised travelers need to be familiar with the immunosuppressive effects resulting from these medications.^22^

The purposes of travel and destinations of immunocompromised travelers were generally similar to the overall traveling population in our study. Leisure travel, including to regions of low socioeconomic development, was common in both groups. Destination countries were similar in both groups, with India being the most popular destination overall. However, there were some notable differences between the itineraries of immunocompromised and immunocompetent travelers. Solid-organ transplant recipients were less likely to travel to countries with low U.N. human development indices and more likely to engage in cruise ship travel, perhaps reflecting more caution among this group of travelers. VFR travel was particularly common among HIV-infected travelers, and HIV-infected travelers were more frequently visiting countries in Africa, including South Africa, Ghana, and Kenya. Immunocompromised people traveled to countries with malarious areas at similar frequencies to immunocompetent travelers but were less likely to be prescribed malaria chemoprophylaxis. This may reflect the pursuit of more cautious itineraries, or alternatively, concern about drug–drug interactions.

Vaccine receipt differed between immunocompromised and immunocompetent travelers. Although many immunocompromised travelers were visiting countries with yellow fever virus transmission, a lower proportion of immunocompromised than immunocompetent travelers received the yellow fever vaccine. This likely reflects the fact that immunosuppression is a contraindication for yellow fever vaccination, but also may suggest more caution regarding itinerary choice among immunocompromised travelers, who may choose to travel to areas without yellow fever when traveling to countries such as Peru and Brazil, where yellow fever virus circulation is not uniformly endemic. The majority of the immunocompromised travelers in this study who received the yellow fever vaccine were HIV-positive people with CD4 count ≥ 500 cells/mm^3^; this practice is consistent with CDC guidelines, which consider asymptomatic HIV infection and HIV infection with CD4 count of 200–499 cells/mm^3^ to be a precaution but not a contraindication for yellow fever vaccine administration.^9^ Many immunocompromised and immunocompetent travelers in this study refused influenza vaccination. Some studies have evaluated the immunogenicity of influenza vaccine in HIV-infected individuals,^23^–^25^ but in general, there are few data on immune responsiveness to travel vaccines in the various subsets of immunocompromised travelers. More research in this area is needed given the frequency of travel in this population.

Our findings identify a few areas where the care of immunocompromised travelers might be improved. First, immunocompromised travelers in this study were seen a median of 25 days before departure; this indicates that many immunocompromised travelers did not have enough time to complete certain travel-related vaccination series, such as for rabies and Japanese encephalitis. Strategies to optimize the timing of pretravel preparation for immunocompromised travelers are important; incorporating routine questioning and planning regarding travel into the primary-care visits of immunocompromised people may be one approach. Immunocompromised business travelers would be a particularly important target for routine pretravel planning, since last-minute travel is more common among business travelers.^12^ Another potential area for improvement is the uptake of influenza vaccine among immunocompromised travelers. Influenza vaccination is universally recommended in the United States for people > 6 months of age,^26^ and the pretravel consultation represents an opportunity to update adult vaccines for all people.^27^ Immunocompromised people are at particular risk of influenza-related complications,^28^ so more effective strategies to educate immunocompromised people about influenza and increased vaccine uptake are needed. Notably, we found that cruise ship travel was favored by certain immunocompromised travelers. Outbreaks of respiratory infections and gastroenteritis have been reported on cruise ships^29^–^31^ and immunocompromised travelers should be counseled by their health-care providers about strategies for avoiding illness in such settings, such as handwashing. Finally, many travelers in this study were traveling to destinations with limited medical infrastructure; a reliable supply of medications and purchase of medical evacuation insurance should be considered for all immunocompromised travelers.

Our study has a few notable limitations. Data were captured by practicing clinicians, but certain details regarding the degree of immune compromise were lacking and hence a large number of people could not be fully classified for the purposes of this study. The full details of the clinical decision-making process were also not available. Our study does not consider people with less severe degrees of immunosuppression, including advanced chronic kidney disease, cirrhosis of the liver, diabetes, older age, and other chronic debilitating medical conditions. GTEN sites include a variety of practice settings, many of which specialize in travel medicine; although we anticipate that immunocompromised travelers may be more likely to seek pretravel care at specialized clinics, the characteristics of immunocompromised travelers seen in other settings may differ from those of people in our study. In particular, immunocompromised travelers traveling to areas of higher development may more commonly seek pretravel consultation in primary care settings.

In conclusion, our results indicate that immunocompromised travelers are pursuing itineraries that are largely similar to those of immunocompetent travelers. Pretravel preparation of the immunocompromised traveler is complex and requires expertise in vaccination, drug interactions, and the nature of various immunocompromising conditions. A strong collaboration between travel-medicine specialists, primary-care doctors, and specialist physicians caring for immunocompromised people is needed to help them make appropriate selections on itinerary, vaccinations, and chemoprophylaxis.

## Figures and Tables

**Table 1:** Immunosuppressive conditions among immunocompromised travelers in Global TravEpiNet*

Immunosuppressive condition	Travelers (*N* = 486)
Immunosuppressive medication	202
Corticosteroids	77
TNF inhibitors	73
Methotrexate	54
Calcineurin inhibitor	65
Sirolimus	7
Mycophenolate mofetil	47
Antimetabolites	47
Alkylating agents	1
Cancer chemotherapy	6
Leflunomide	4
Hydroxyurea	6
Rituximab	4
Ustekinumab	1
HIV infection	110
CD4 count > 500	71
CD4 count 200–500	22
CD4 count < 200	9
Solid-organ transplant	61
Splenectomy	47
Hematological malignancy	27
Sickle cell anemia	9
Stem cell or bone marrow transplant	7
Neutropenia	5
Hypogammaglobulinemia	4
History of thymectomy or thymus disease	1

TNF = tumor necrosis factor.

* Patients can have ≥ 1 immunosuppressive condition or be taking more than one immunosuppressive medication.

**Table 2:** Demographic and travel-related characteristics of immunocompromised travelers compared with immunocompetent travelers‡

	Immunocompromised travelers	Immunocompetent travelers	*P* value*
Number (% of total)	486 (1.6)	30,702 (98.4)	–
Age (median, range)	46 (1.5–83)	35 (0–94)	< 0.0001
Female	241 (50%)	16,672 (54%)	0.11
Destination (UNHDI classification)†			0.02
Low human development	138 (28%)	9,507 (31%)	–
Medium human development	242 (50%)	14,852 (48%)	–
High human development	84 (17%)	5,407 (18%)	–
Very high human development	22 (5%)	936 (3%)	–
Days to departure (median, range)	25 (0–424)	25 (0–564)	0.0004
Duration of travel (days; median, range)	14 (2–700)	14 (0–9999)	0.05
Purpose of travel§			
Leisure	288 (59%)	16,563 (54%)	0.01
Business	80 (16%)	5,711 (19%)	0.05
VFR	62 (13%)	2,898 (9%)	0.29
Non-medical service work	13 (3%)	2,253 (7%)	0.002
Missionary work	20 (4%)	1,738 (6%)	0.36
Adventuring	15 (3%)	1,678 (6%)	0.003
Accommodations§			
Camping	21 (4%)	2,213 (7%)	0.01
Dormitory or hostel	41 (8%)	5,191 (17%)	< 0.0001
Home stay with relatives	92 (19%)	4,516 (15%)	0.07
Hotel	353 (73%)	21,239 (69%)	0.19
Cruise	38 (8%)	1,240 (4%)	< 0.0001
Taking a medication currently	459 (94%)	17,584 (57%)	< 0.0001
Number of medications per person (median, range)	3 (0–11)	1 (0–15)	< 0.0001

UNHDI = United Nations Human Development Index; VFR = visiting friends and relatives.

* *P* value calculated via random intercept model.

† Travelers can travel to ≥ 1 destination.

‡ All percentages are column percentages unless otherwise stated.

§ Travelers could choose more than one purpose of travel or more than one accommodation.

**Table 3:** Travel characteristics of sub-groups of immunocompromised travelers

	HIV		Solid-organ transplant (*N* = 61)	TNF inhibitors (*N* = 73)
	CD4 ≥ 500 cells/mm^3^ (*N* = 71)	CD4 < 500 cells/mm^3^ (*N* = 39)		
Top destinations	Kenya (8%)	South Africa (15%)	India (15%)	India (18%)
	Ghana (7%)	Ghana (10%)	China (10%)	Thailand (10%)
	Guinea (7%)	India (8%)	Italy (8%)	Kenya (7%)
	South Africa (7%)	Peru (8%)	Greece (7%)	Cambodia (7%)
		Senegal (8%)		
		Zambia (8%)		
Travel to region with low human development (UNHDI classification)*	33 (46%)	20 (51%)	5 (8%)	23 (32%)
Purpose of travel†				
Leisure	36 (51%)	18 (46%)	31 (51%)	41 (56%)
Business	14 (20%)	6 (15%)	12 (20%)	17 (23%)
VFR	16 (23%)	16 (41%)	11 (18%)	2 (3%)
Non-medical service work	2 (3%)	0	1 (2%)	2 (3%)
Missionary work	1 (1%)	0	6 (10%)	1 (1%)
Adventuring	3 (4%)	1 (3%)	0	2 (3%)
Days before departure visited travel clinic (median, range)	21 (0–257)	22 (4–98)	22 (0–252)	25 (0–353)

* United Nations Human Development Index (UNHDI).

† Visiting friends and relatives (VFR).

## References

[1] International Trade Administration (2013). Office of Travel and Tourism Industries, U.S. Department of Commerce. http://tinet.ita.doc.gov/outreachpages/outbound.general_information.outbound_overview.html.

[2] United Nations World Tourism Organization Press Release No. PR120020, January 2012. http://media.unwto.org/en/press-release/2012-01-16/international-tourism-reach-one-billion-2012.

[3] Organ Procurement and Transplantation Network U.S. transplants performed January–March 2014. http://optn.transplant.hrsa.gov/data/.

[4] Chen M, Rhodes PH, Hall IH, Kilmarx PH, Branson BM, Valleroy LA, Centers for Disease Control and Prevention (CDC) (2012). Prevalence of undiagnosed HIV infection among persons aged ≥ 13 years—national HIV surveillance system, United States, 2005–2008. Morb Mortal Wkly Rep.

[5] Uslan DZ, Patel R, Virk A (2008). International travel and exposure risks in solid-organ transplant recipients. Transplantation.

[6] Kemper CA, Linett A, Kane C, Deresinski SC (1995). Frequency of travel of adults infected with HIV. J Travel Med.

[7] Kemper CA, Linett A, Kane C, Deresinski SC (1997). Travels with HIV: the compliance and health of HIV-infected adults who travel. Int J STD AIDS.

[8] Salit IE, Sano M, Boggild AK, Kain KC (2005). Travel patterns and risk behaviour of HIV-positive people travelling internationally. CMAJ.

[9] (2011). CDC Health Information for International Travel 2012.

[10] Roukens AH, van Dissel JT, de Fijter JW, Visser LG (2007). Health preparations and travel-related morbidity of kidney transplant recipients traveling to developing countries. Clin Transplant.

[11] Wieten RW, Leenstra T, Goorhuis A, van Vugt M, Grobusch MP (2012). Health risks of travelers with medical conditions—a retrospective analysis. J Travel Med.

[12] LaRocque RC, Rao SR, Lee J, Ansdell V, Yates JA, Schwartz BS, Knouse M, Cahill J, HAgmann S, Vinetz J, Connor BA, Goad JA, Oladele A, Alvarez S, Stauffer W, Walker P, Kozarsky P, Franco-Paredes C, Dismukes R, Rosen J, Hynes NA, Jacquerioz F, McLellan S, Hale D, Sofarelli T, Schoenfeld D, Marano N, Brunette G, Jentes ES, Yanni E, Sotir MJ, Ryan ET, Global Travel Epinet Consortium (2012). Global TravEpiNet: a national consortium of clinics providing care to international travelers—analysis of demographic characteristics, travel destinations, and pretravel healthcare of high-risk US international travelers, 2009–2011. Clin Infect Dis.

[13] United Nations Development Programme (2011). Human Development Report.

[14] Rao SR, LaRocque RC, Jentes ES, Hagmann SHF, Ryan ET, Han PV, Kleinbaum DG (2014). Comparison of methods for clustered data analysis in a non-ideal situation: results from an evaluation of predictors of yellow fever vaccine refusal in the Global TravEpiNet (GTEN) consortium. Int J Stat Med Res.

[15] Keane J, Gershon S, Wise RP, Mirabile-Levens E, Kasznica J, Schwieterman WD, Siegel JN, Braun MM (2001). Tuberculosis associated with infliximab, a tumor necrosis factor alpha-neutralizing agent. N Engl J Med.

[16] Winthrop KL, Yamashita S, Beekmann SE, Polgreen PM, Infectious Diseases Society of America Emerging Infections Network (2008). Mycobacterial and other serious infections in patients receiving anti-tumor necrosis factor and other newly approved biologic therapies: case finding through the emerging infections network. Clin Infect Dis.

[17] Zanger P, Kotter I, Kremsner PG, Gabrysch S (2012). Tumor necrosis factor alpha antagonist drugs and leishmaniasis in Europe. Clin Microbiol Infect.

[18] Pena-Sagredo JL, Farinas MC, Perez-Zafrilla B, Cruz-Valenciano A, Crespo M, Joven-Ibanez B, Riera E, Manero-Ruiz FJ, Chalmeta I, Hernandez MV, Rodriguez-Gomez M, Maiz O, Lopez R, Cobo T, Pita J, Carmona L, Gonzales-Gay MA, BIOBADASER and EMECAR Groups (2009). Non-typhi *Salmonella* infection in patients with rheumatic diseases on TNF-alpha antagonist therapy. Clin Exp Rheumatol.

[19] Salemi S, Picchianti-Diamanti A, Germano V, Donatelli I, Di Martino A, Facchini M, Nisini R, Biselli R, Ferlito C, Podesta E, Cappella A, Milanetti F, Rossi F, Amodeo R, Tabacco F, Di Rosa R, Lagana B, D Amelio R (2010). Influenza vaccine administration in rheumatoid arthritis patients under treatment with TNFalpha blockers: safety and immunogenicity. Clin Immunol.

[20] Kaine JL, Kivitz AJ, Birbara C, Luo AY (2007). Immune responses following administration of influenza and pneumococcal vaccines to patients with rheumatoid arthritis receiving adalimumab. J Rheumatol.

[21] Rubin LG, Levin MJ, Ljungman P, Davies EG, Avery R, Tomblyn M, Bousvaros A, Dhanireddy S, Sung L, Keyserling H, Kang I, Infectious Diseases Society of America (2014). 2013 IDSA clinical practice guideline for vaccination of the immunocompromised host. Clin Infect Dis.

[22] Orenstein R (2005). Travel in patients receiving TNF-alpha inhibitors. Travel Med Infect Dis.

[23] Curtis DJ, Muresan P, Nachman S, Fenton T, Richardson KM, Dominguez T, Flynn PM, Spector SA, Cunningham CK, Bloom A, Weinberg A (2015). Characterization of functional antibody and memory B-cell responses to pH1N1 monovalent vaccine in HIV-infected children and youth. PLoS One.

[24] Berger CT, Greiff V, Mehling M, Fritz S, Meier MA, Hoenger G, Conen A, Recher M, Battegay M, Reddy ST, Hess C (2015). Influenza vaccine response profiles are affected by vaccine preparation and preexisting immunity, but not HIV infection. Hum Vaccin Immunother.

[25] George VK, Pallikkuth S, Parmigiani A, Alcaide M, Fischl M, Arheart KL, Pahwa S (2015). HIV infection worsens age-associated defects in antibody responses to influenza vaccine. J Infect Dis.

[26] Centers for Disease Control and Prevention (CDC) (2013). Prevention and control of seasonal influenza with vaccines. recommendations of the Advisory Committee on Immunization Practices—United States, 2013–2014. MMWR Recomm Rep.

[27] LaRocque RC, Jentes ES (2011). Health recommendations for international travel: a review of the evidence base of travel medicine. Curr Opin Infect Dis.

[28] Mauskopf J, Klesse M, Lee S, Herrera-Taracena G (2013). The burden of influenza complications in different high-risk groups: a targeted literature review. J Med Econ.

[29] Centers for Disease Control and Prevention (CDC) (2005). Cruise-ship–associated Legionnaires disease, November 2003–May 2004. Morb Mortal Wkly Rep.

[30] Wikswo ME, Cortes J, Hall AJ, Vaughan G, Howard C, Gregoricus N, Cramer EH (2011). Disease transmission and passenger behaviors during a high morbidity norovirus outbreak on a cruise ship, January 2009. Clin Infect Dis.

[31] Miller JM, Tam TW, Maloney S, Fukuda K, Cox N, Hockin J, Kertesz D, Klimov A, Cetron M (2000). Cruise ships: high-risk passengers and the global spread of new influenza viruses. Clin Infect Dis.

